# The Role of ^18^F-FDG PET/CT and MRI in Assessing Pathological Complete Response to Neoadjuvant Chemotherapy in Patients with Breast Cancer: A Systematic Review and Meta-Analysis

**DOI:** 10.1155/2016/3746232

**Published:** 2016-02-15

**Authors:** Qiufang Liu, Chen Wang, Panli Li, Jianjun Liu, Gang Huang, Shaoli Song

**Affiliations:** Department of Nuclear Medicine, Ren Ji Hospital, Shanghai Jiao Tong University, School of Medicine, 160 Pujian Road, Shanghai 200127, China

## Abstract

*Purpose*. We performed this meta-analysis to determine the utilities of ^18^F-FDG PET/CT and MRI in assessing the pathological complete response (pCR) after neoadjuvant chemotherapy (NAC) in the same cohort of patients with breast cancer.* Methods*. Two reviewers systematically searched on PubMed, Scopus, and Springer (from the beginning of 1992 to Aug. 1, 2015) for the eligible articles. Heterogeneity, pooled sensitivity and specificity, positive likelihood ratio, negative likelihood ratio, and the summary receiver operating characteristic (SROC) curve were calculated to estimate the diagnostic efficacy of ^18^F-FDG PET/CT and MRI.* Results*. A total of 6 studies including 382 pathologically confirmed patients were eligible. The pooled sensitivity and specificity of ^18^F-FDG PET/CT were 0.86 (95% CI: 0.76–0.93) and 0.72 (95% CI: 0.49–0.87), respectively. Pooled sensitivity and specificity of MRI were 0.65 (95% CI: 0.45–0.80) and 0.88 (95% CI: 0.75–0.95), respectively. The area under the SROC curve of ^18^F-FDG PET/CT and MRI was 0.88 and 0.84, respectively.* Conclusion*. Study indicated that ^18^F-FDG PET/CT had a higher sensitivity and MRI had a higher specificity in assessing pCR in breast cancer patients. Therefore, the combined use of these two imaging modalities may have great potential to improve the diagnostic performance in assessing pCR after NAC.

## 1. Introduction

Neoadjuvant chemotherapy (NAC) has become a standard therapy for patients with locally advanced or inflammatory breast cancer these years. One of the major advantages of neoadjuvant chemotherapy is that reduction of the tumor size and downstaging of tumor burden may facilitate the successful performance of breast-conserving surgery instead of mastectomy [1, 2]. In addition, it can also evaluate the therapy response to find the right time to do the operation and adjust the treatment plan in case of an unfavorable tumor response at an early stage [3]. Results from several studies have demonstrated superior disease-free survival and overall survival in patients who achieve a pCR (pathological complete response) [4, 5]. Hence, achieving pCR is an important treatment objective for patients with breast cancer. However, we cannot accurately observe the pCR until the definitive breast surgery, and this will always lead to deficient or excessive chemotherapy and inappropriate surgery decision-making for patients before surgery. Therefore, to find an effective method to evaluate the pCR and to avoid unnecessary surgery option before surgery is crucial in the management of these patients.

Noninvasive imaging tools that could monitor the response to NAC are particularly attractive. Magnetic resonance imaging (MRI) and ^18^F-fluorodeoxyglucose positron emission tomography/computed tomography (^18^F-FDG PET/CT) are increasingly being used to screen and monitor the response to NAC in breast cancer [6, 7]. In case of locally advanced breast cancer, MRI has the potential to select those patients that are eligible for conservative surgical treatment after NAC [8, 9]. In addition, as noted by Buchbender and colleagues, PET/MRI with lower radiation exposure and joining all the benefits of morphologic and functional MRI information and metabolic PET information may be most useful in setting of evaluation for suspected tumor recurrence, response to neoadjuvant therapy, and prognosis [10]. But the fact is that PET/MRI has not been widely used in clinical practice. ^18^F-FDG PET/CT and MRI in predicting pCR had been previously reported [7, 11]. Due to different types of pathology and sample sizes used in each study, the diagnostic performance of ^18^F-FDG PET/CT and MRI has varied dramatically in breast cancer [12–14]. More importantly, most published articles evaluated the two modalities separately, not in the same group of patients. Thus, we conducted this meta-analysis to evaluate the overall diagnostic performance of ^18^F-FDG PET/CT and MRI in assessing pCR after NAC in the same cohort of patients with breast cancer.

## 2. Methods

### 2.1. Literature Search

We searched on PubMed, Scopus, and Springer (from the beginning of 1992 to Aug. 1, 2015), using “positron emission tomography OR positron emission tomography/computed tomography OR PET OR PET/CT OR PET-CT”, “breast neoplasm OR breast carcinoma OR breast cancer OR breast tumor OR breast tumor”, “response OR prediction”, and “MR OR MRI” as keywords. Certain filters were used for EMBASE according to inclusion criteria described in the following. Two reviewers independently screened titles and abstracts to select potential articles and then further examined full text articles of all potentially eligible citations. We also screened the reference list of retrieved studies for any additional publications.

### 2.2. Eligibility Criteria

The inclusion criteria were as follows: (1) patients should receive both ^18^F-FDG PET/CT scan and MRI examination before and during (after) NAC; (2) studies should be prospective or retrospective; (3) at least 10 patients were included in the article; (4) the studies should contain raw data, such as TP, FP, TN, and FN; (6) the gold standard of pCR should be complete absence of residual invasive tumor cells irrespective of carcinoma in situ or have no residual tumors and no metastatic lymph nodes; (7) for MRI assessment, complete response (CR) could be defined as having no significant enhancement on postchemotherapy MR images or at least a 30% decrease in the maximal diameter (*D*_max_) or volume of the tumor; (8) PET/CT assessment parameters could be SUV or SUV_max_ or pSUV. CR was defined as having completely no ^18^F-FDG uptake of the tumor or at least a reduction of 50% in the SUV or SUV_max_ or pSUV compared with pre-NAC. The criteria were confirmed by two other reviewers (L. P, S. S). Duplicated articles, reviews, case reports, conference abstracts, animals, and cells studies and other nonrelated articles were excluded.

### 2.3. Quality Assessment of the Studies

Two reviewers assessed methodological quality of eligible studies independently by using the QUADAS-2 (quality assessment of diagnosis accuracy study), which is a newly revised quality assessment tool specifically developed for systematic reviews of diagnostic accuracy studies [15]. It contains 4 aspects: patient selection, index test, reference standard, and flow and timing. Each domain was assessed in terms of the risk of bias, and the first three were also assessed in terms of concerns regarding applicability. Signaling questions are used to classify studies as having high, low, or unclear risk. Reasons for classifying some articles into high risk categories were as follows: the criteria were not clear, patients were not collected in consecutive order, the study did not indicate the parameters, images were not explained blind to pathology, inaccurate reference standard was used to classify the patients, and not all the patients were included in analysis in the study.

### 2.4. Data Extraction

For each eligible study, we extracted the following information: first author, county, year of publication, patients' demographic and clinical characteristics, therapeutic interventions, scan time of ^18^F-FDG PET/CT and MRI, reference standard, and number of responders and nonresponders results. True positive (TP), false positive (FP), false negative (FN), and true negative (TN) were obtained from the ^18^F-FDG PET/CT and MRI scan results after they had been compared with the pathological results. Data extraction was done independently by two reviewers, and in case of any discrepancies, consensus was reached to solve them.

### 2.5. Data Synthesis and Statistical Analysis

For each study, a 2-by-2 contingency table was constructed to classify patients into 1 of 4 groups: true positives, true negatives, false positives, and false negatives. By using the tables, we calculated the true-positive rate (TPR; sensitivity), the false-positive rate (FPR; 1 − specificity), the positive predictive value (PPV), and the negative predictive value (NPV).

The heterogeneity among different studies was analyzed using Chi-squared test. And it was assessed by forest plot where *Q* and *I* ^2^ statistics were presented. If there was heterogeneity, which was defined as *I* ^2^ > 50%, the random effects model (REM) was selected; conversely, the fixed effects model (FEM) was selected. Threshold effect was an important source of heterogeneity. To judge whether the threshold effect exists, the Spearman correlation coefficients (between the logit of sensitivity and logit of specificity) for PET/CT and MRI were calculated. If *P* > 0.05, there was no threshold effect. And the forest plot and summary receiver operating characteristic (SROC) curve were drawn using the bivariate mixed effects models [16]. The area under the curve (AUC) of the SROC was calculated to measure the performance of ^18^F-FDG PET/CT and MRI. We also calculated the Youden index (^*∗*^ *Q*), which is the best statistical method to reflect the diagnostic value [17]. Then *Z*-test was performed to find whether the sensitivity, specificity, and ^*∗*^ *Q* index of one modality are significantly different from the other one. All analyses were carried out using Stata 12.0 and Meta-DiSc 14.0.

The publication bias of all included studies was analyzed by using “Deek” funnel plot. Statistical calculation and analysis were performed using Stata 12.0 (StataCorp, College Station, TX, USA).

## 3. Results

### 3.1. Study Selection

**Figure 1:**
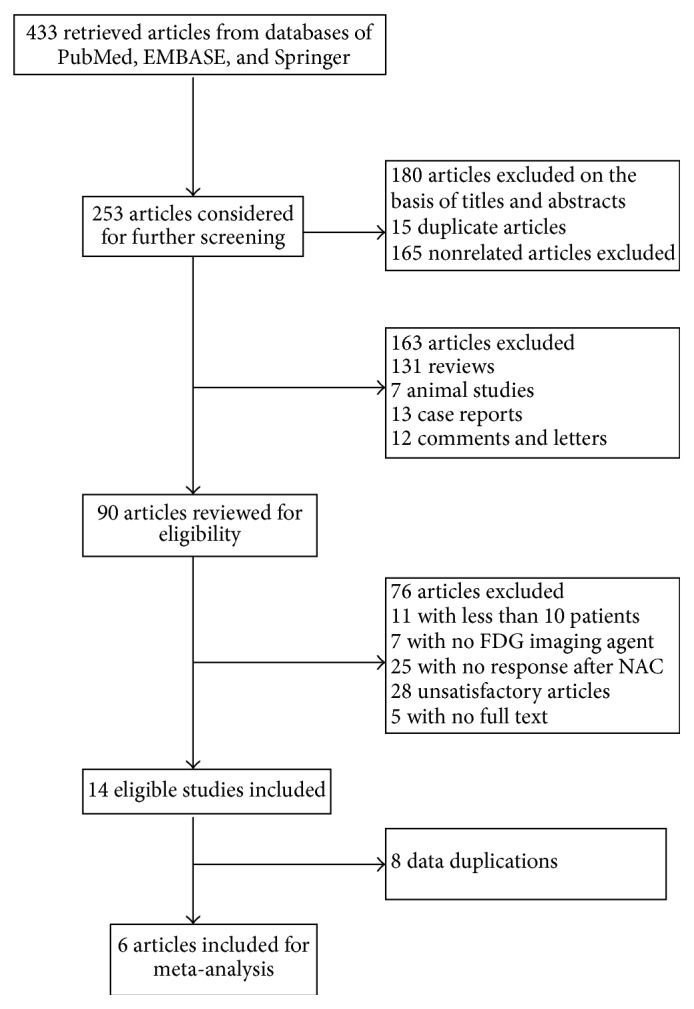
Flowchart of the literature search in the meta-analysis.

Systematic search yielded a total of 433 studies from the databases of PubMed, EMBASE, and Springer. After reviewing the titles and abstracts, 90 articles were considered as potential eligible candidates for inclusion. After in-depth reading, we excluded 76 articles since they have not met our eligibility criteria. Of the remaining 14 eligible studies, there were 8 data duplications. Thus, we have included 6 articles in our current meta-analysis [18–23]. A diagram schematizing the selection process is presented in Figure 1.

### 3.2. Study Description and Patients Characteristics

Table 1 showed that a total of 382 patients were included, and within all studies included, there were 1 from the USA, three from Korea, one from Japan, and 1 from Netherlands. Of the 6 articles, three were prospective studies and the rest of them were retrospective. The measuring parameters of ^18^F-FDG PET/CT and MRI were varied. For ^18^F-FDG PET/CT, five of the 6 included articles chose SUV_max_ [18, 20–23]; one study used peak-standardized uptake values (pSUV) within the ROIs [19]. For MRI, five studies measured the diameter of tumor while 1 study analyzed the data extracted from dynamic gadolinium concentration curves [18–23]. All the 6 articles used pathologic response as the golden standard [18–23]. Additionally, most of them defined pCR as “no recognizable invasive tumor cells were encountered.” Details of the 6 studies were shown in Table 2. One of the included studies has evaluated diagnostic value of ultrasound, PET/CT, MRI, or CT on predicting pCR after performing NAC on breast cancer patients, and we only extracted data of PET/CT and MRI for our meta-analysis [19].

### 3.3. Quality Assessment

**Figure 2:**
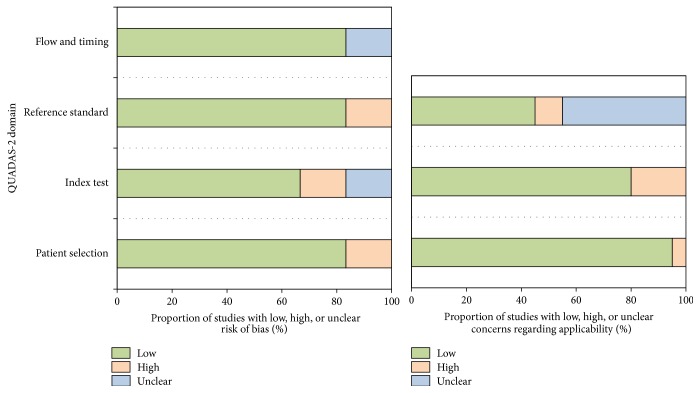
Proportion of studies with low, high, or unclear risk of bias. Proportion of studies with low or unclear concerns regarding applicability.

The detailed information and scores regarding the quality assessment are displayed in Figure 2. In general, the results of QUADAS-2 tool showed that the qualities of included studies were satisfying and eligible.

### 3.4. Heterogeneity Test

**Figure 3:**
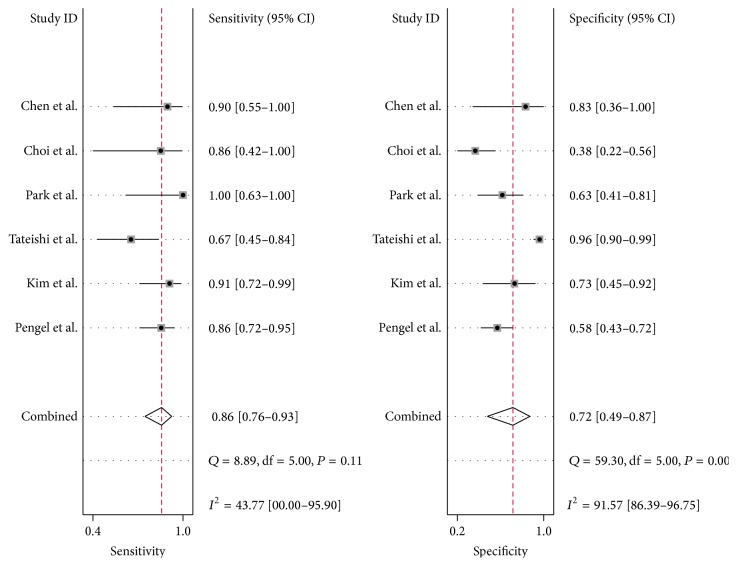
Forest plot of the ^18^F-FDG PET/CT of 6 included studies. The size of the square plotting reflects the study weight. Horizontal lines are the 95% confidence intervals.

**Figure 4:**
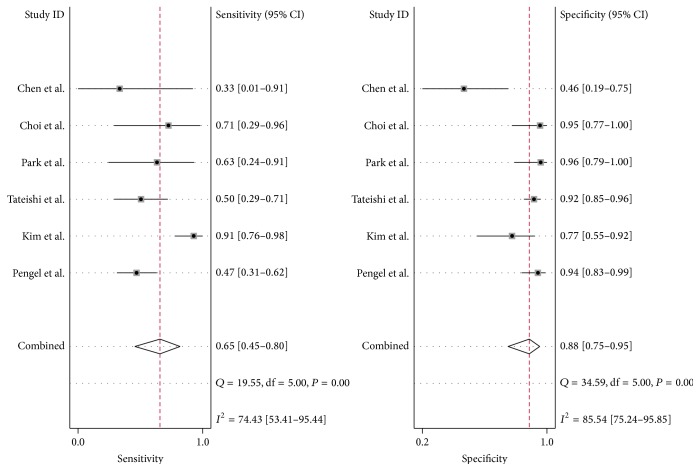
Forest plot of the MRI of 6 included studies. The size of the square plotting reflects the study weight. Horizontal lines are the 95% confidence intervals.

The results showed that there were statistical heterogeneity of ^18^F-FDG PET/CT (sensitivity, *Q* value = 8.89, *I* ^2^ = 43.77%; specificity, *Q* value = 59.30, *I* ^2^ = 91.57%) and MRI (sensitivity, *Q* value = 19.55, *I* ^2^ = 74.43%; specificity, *Q* value = 34.59, *I* ^2^ = 85.54%) (Figures 3 and 4). Considering the heterogeneity indicated by *I* ^2^, bivariate mixed effects model was chosen to synthesize the ROC curves.

The Spearman correlation coefficient and *P* value of PET/CT and MRI were 0.086 (*P* = 0.872, *P* > 0.05) and −0.314 (*P* = 0.544, *P* > 0.05), respectively. These results showed that there were no threshold effects in this meta-analysis.

**Figure 5:**
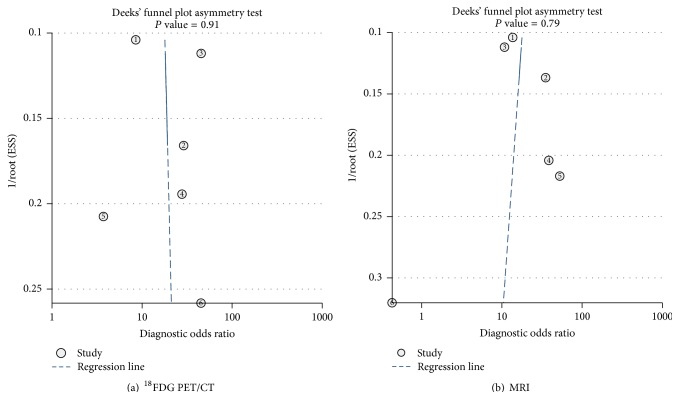
(a) Publication bias of ^18^F-FDG PET/CT using Deek's funnel plot. (b) Publication bias of MRI using Deek's funnel plot.

The publication bias was shown in Figure 5. The publication bias of both ^18^F-FDG PET/CT and MRI was insignificant (*P* = 0.91, *P* = 0.79, resp.). These results indicated that there was no significant publication bias.

### 3.5. Performance of ^18^F-FDG PET/CT and MRI in Assessing Response to Preoperative NAC

**Figure 6:**
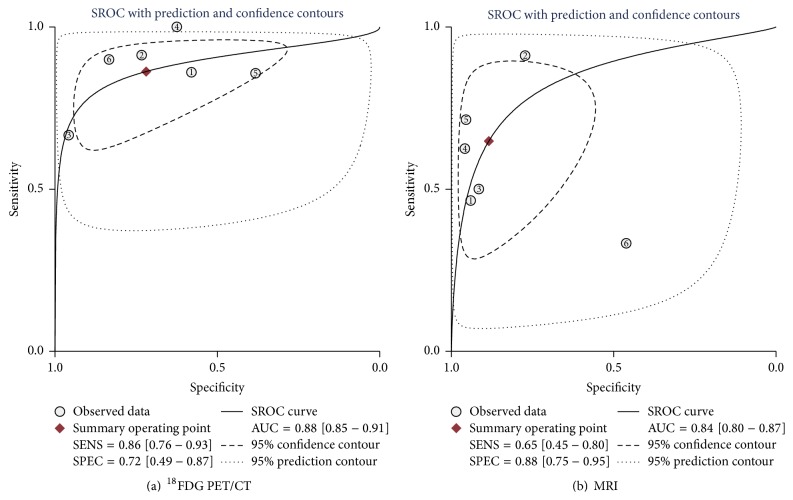
(a) SROC for NAC response prediction in primary breast cancer by ^18^F-FDG PET/CT. (b) SROC for NAC response prediction in primary breast cancer by MRI.

The pooled sensitivity of ^18^F-FDG PET/CT and MRI was 0.86 (95% CI, 0.76–0.93) and 0.65 (95% CI, 0.45–0.80), respectively. The sensitivity of ^18^F-FDG PET/CT was higher than that of MRI (*P* < 0.05). And the specificity of ^18^F-FDG PET/CT and MRI was 0.72 (95% CI, 0.49–0.87) and 0.88 (95% CI, 0.75–0.95), respectively (Figures 3 and 4). The specificity of MRI was higher than that of ^18^F-FDG PET/CT (*P* < 0.05). For ^18^F-FDG PET/CT, positive likelihood ratio, negative likelihood ratio, and diagnostic odds ratio were 3.1 (95% CI, 1.6–5.9), 0.19 (95% CI, 0.11–0.32), and 16 (95% CI, 7–37), respectively. While for MRI, positive likelihood ratio, negative likelihood ratio, and diagnostic odds ratio were 5.6 (95% CI, 2.5–0.91), 0.40 (95% CI, 0.24–0.65), and 14 (95% CI, 5–40), respectively. SROC curves showed that the AUC of ^18^F-FDG PET/CT and MRI were 0.88 (95% CI, 0.85–0.91) and 0.84 (95% CI, 0.80–0.87), respectively (Figure 6). The Youden index (^*∗*^ *Q*) estimates for ^18^F-FDG PET/CT and MRI were 0.82 and 0.77, respectively. And the ^*∗*^ *Q* index of ^18^F-FDG PET/CT was higher than that of MRI (*P* < 0.05).

## 4. Discussion

Breast cancer response to NAC has traditionally been assessed by conventional imaging modalities such as mammogram and ultrasound. These anatomical imaging modalities sometimes have difficulties in differentiating fibrosis from residual tumors; thus they are of limited use for monitoring the treatment response. Nowadays, ^18^F-FDG PET/CT and enhanced-MRI are two imaging modalities mostly used in clinical practice. ^18^F-FDG PET/CT can differentiate changes in tumor glucose metabolism before morphologic changes. The decrease in ^18^F-FDG uptakes in tumors after chemotherapy is an indicator to assess the treatment response. Enhanced-MRI can provide information on lesion microvasculature and depict changes in the physiologic characteristics of tumors. Several studies had addressed the role of ^18^F-FDG PET/CT and MRI in assessing the early response of breast tumors to chemotherapy separately in different cohort patients.

In this study, we systematically calculated the predictive performance of ^18^F-FDG PET/CT and MRI in the same cohort of patients with breast cancer. The results showed that ^18^F-FDG PET/CT had a higher sensitivity when compared with MRI. This may contribute to the following reasons: firstly, ^18^F-FDG PET/CT exhibited unique advantages to offer an earlier metabolic response prediction than morphologic images. But ^18^F-FDG is a nonspecific tracer and also accumulates in sites of inflammation. Chemotherapy induced therapeutic effects often caused apoptosis, necrosis, and inflammation [24, 25]. That caused false positive in ^18^F-FDG PET imaging and made it difficult in image interpretation after NAC. Secondly, for most breast cancer patients with advanced stages, chemotherapy always caused tumor shrinkage rather than disappearing. When the reduction rate of *D*_max_ reached the specified threshold value or the lesion was no longer enhanced or completely gone, it was a complete response on MRI.

For MRI, our study results were similar in conclusion with the study by Wu et al. (sen.: 68%, spe.: 91% versus sen.: 65%, spe.: 88%) [11]. In another meta-analysis study focused on residual detection of breast cancer after NAC, the sensitivity and specificity of contrast enhanced-MRI were 54% and 87%, respectively [26]. Compared with these two similar studies, our results showed higher sensitivity and specificity [27, 28]. SUV cut-off value may be another significant factor influencing the results. Mghanga et al. studied the diagnostic performance of PET/CT in breast cancer patients who underwent NAC. Study results showed a lower sensitivity (80.5% versus 86%) and a higher specificity (78.8% versus 72%) compared with ours [28]. Causes of the difference mainly because of SUV cut-off value in their study ranged from 40% to 88%, but ours were not less than 50%.

The integrated whole-body PET/MRI that is of lower radiation exposure and can provide high-resolution anatomical, morphological, molecular information particularly for soft tissues has attracted more and more attention in recent years. But there is limited data which has been published on the role of PET/MRI in the assessment of response after the NAC. And PET/MRI imaging in oncologic patient population is mainly applied to cover single organ region or whole-body tumor staging, restaging, and metastasis screening [29, 30]. For the high NPV (PET component) and the high specificity (MRI component), PET/MRI hold the promise to improve therapy-response evaluation [10]. For now, prospective studies are needed to demonstrate if there is improved diagnostic accuracy and cost-effectiveness of combined ^18^F-FDG PET/MR compared to ^18^F-FDG PET/CT.

As PET/CT has a sensitivity of 86% and MRI has a specificity of 88%, the combined use of these two imaging modalities may have great potential to improve the diagnostic performance in assessing pCR after NAC. But in clinical practice, there were cases that the results of FDG PET/CT and MRI may be different. Considering that PET/CT has higher sensitivity and higher accuracy in TNM staging in clinical practice, PET/CT results may be the most often used information in determining the resection margin.

There were still some limitations of our study. First, considering the limited number of the published studies for MRI and ^18^F-FDG PET/CT in the same cohort of patients, the small cohort of patients for the two imaging modalities may cause heterogeneity. Second, the heterogeneity among studies might also come from various types and stages of breast cancer which are included in our meta-analysis. Finally, we acknowledge other potential limitations including selector bias, which was brought about by selection, publication, and verification of the studies.

## 5. Conclusion

Study indicated that ^18^F-FDG PET/CT had a higher sensitivity and MRI had a higher specificity in assessing pCR in breast cancer patients. Therefore, the combined use of these two imaging modalities may have great potential to improve the diagnostic performance in assessing pCR after NAC.

## Supplementary Material

Table 3: The Signaling questions that were used for judging the risk of bias and applicability of the included studies. Table 4: The raw datas including numbers of true-positive (TP), false-positive (FP), true-negative (TN), and false-negative (FN) results for each modality for both patient.

## Figures and Tables

**Table 1:** Characteristics of included studies for detecting pathologic response to neoadjuvant chemotherapy.

Author	County	Year	Study design	Number of patients	Mean age	Type of NAC	Cycles of NAC	Evaluation time	Endpoint	pCR/npCR	Evaluation index	Sen.
Pengel et al. [23]	Netherlands	2014	Pro.	93	47.8	Trastuzumab-based regimen	3 or 6	B/A (1 or 3 cycles)	pCR	43/50	MRI PET/CT	0.465 0.860
Kim et al. [22]	Korea	2014	Retro.	59	46.6	NR	3 or 6	B/A (3 or 6 cycles)	Miller and Payne system	34/22 23/15	MRI PET/CT	0.912 0.913
Tateishi et al. [21]	Japan	2012	Retro.	142	57	5-Fluorouracil, epirubicin, cyclophosphamide, and so forth	4	B/A (2 cycles)	pCR	24/118	MRI PET/CT	0.50 0.667
Park et al. [20]	Korea	2011	Retro.	32	45	Doxorubicin and docetaxel	3 or 6	B/A (18–22 days)	pCR	8/24	MRI PET/CT	0.625 1
Choi et al. [19]	Korea	2010	Pro.	41	45.1	Adriamycin and cyclophosphamide or docetaxel	3 or 8	B/A (3 or 8 cycles)	pCR	7/34	MRI PET/CT	0.714 0.857
Chen et al. [18]	USA	2004	Pro.	15	44	Anthracycline-based regimen	NR	B/A	pCR	10/6	MRI PET/CT	0.333 0.90

NR: not reported.

B/A: before or after the NAC.

pCR: pathology complete response.

Pro.: prospect. Retro.: retrospect.

**Table 2:** Characteristics of the patients and explanation method of the two evaluating methods (^18^F-FDG PET/CT and MRI).

Author	County	Year	Initial clinical stage	Histology subtype	Receptor status	Parameter of PET/CT	Parameter of MRI	Reconstruction or not	Image interpretation	Cut-off value of PET/CT	Cut-off value of MRI
Pengel et al. [23]	Netherlands	2014	II (49) III (41) IVa (3)	IDC (85) ILC (7) Others (1)	HER-2(+) (25) ER(+)/PR(−) (4) Triple(−) (28)	ROI + SUV_max_	ROI + diameter	Yes	NR	Reduction > 50%	Reduction > 75% (*D*)
Kim et al. [22]	Korea	2014	II (24) III (33) IVa (2)	IDC (54) ILC (1) MC (1)	NR	ROI + SUV_max_	ROI + diameter ROI + volume	Yes	Blind	Reduction > 60.1%	Reduction > 50% (*D*) Reduction > 68.9% (*V*)
Tateishi et al. [21]	Japan	2012	Ia (9) II (95) III (38)	IDC (131) ILC (11)	ER(+) (100) PR(+) (82) HER-2(+) (111)	ROI + SUV_max_	ROI + Kep	Yes	NR	Reduction > 50%	Reduction > 30% (*D*)
Park et al. [20]	Korea	2011	NR	IDC (31) MC (1)	ER(+) (14) PR(+) (13)	ROI + SUV_max_	ROI + diameter	Yes	Blind	Reduction > 50%	Reduction > 30% (*D*)
Choi et al. [19]	Korea	2010	II, III	IDC (36) ILC (2) MC (1) Other (2)	ER(+) (19) PR(+) (15) HER-2(+) (10)	ROI + pSUV	ROI + diameter	NR	Blind	Reduction > 50%	Reduction > 30% (*D*)
Chen et al. [18]	USA	2004	LABC	IDC (12) ILC (3) MC (1)	ER(+) (12) PR(+) (11) HER-2(+) (2)	ROI + SUV_max_	ROI + diameter	Yes	Blind	Reduction > 50%	Reduction > 30% (*D*)

LABC: local advanced breast cancer.

IDC: invasive ductal carcinomas.

ILC: invasive lobular carcinomas.

MC: metaplastic carcinoma.

ER: estrogen receptor.

PR: progesterone receptor.

ROI: region of interest.

*D*: diameter.

*V*: volume.

NR: not reported.
