# Supplementary material for: The Role of 18F-FDG PET/CT and MRI in Assessing Pathological Complete Response to Neoadjuvant Chemotherapy in Patients with Breast Cancer: A Systematic Review and Meta-Analysis
Source: Biomed Res Int. 2016 Feb 15;2016:3746232. doi: 10.1155/2016/3746232 (PMC4770138; doi:10.1155/2016/3746232)
Supplement: Supplementary file 1 — Table 3: The Signaling questions that were used for judging the risk of bias and applicability of the included studies. Table 4: The raw datas including numbers of true-positive (TP), false-positive (FP), true-negative (TN), and false-negative (FN) results for each modality for both patient. [file 3746232.f1.docx]

**Table 3. Risk of Bias and Applicability Judgments in QUADAS-2**

| Domian | Patient Selection | Index Test | Reference Standard | Flow and Timing |
| --- | --- | --- | --- | --- |
| Signaling questions  (Yes, no or unclear) | Was a consecutive or random sample of patients enrolled?  Did the study avoid inappropriate exclusions?  Did the study clearly indicate the patient select criteria? | Did the study clearly indicate the parameters of the imaging methods?  Did the doctors interpret the images without knowledge of the results of the reference standard?  If a threshold was used, was it prespecified? | Was the reference standard clearly defined?  Was the reference standard likely to correctly judge the response to NAC ? | Did all patients receive a reference standard?  Were all patients included in the ananlysis? |
| Risk of bias  (high, low or unclear) | Could the selection of patients have introduced bias? | Could the conduct or interpretation of the images have introduced bias? | Could the reference standard, or its conduct, or its interpretation have  introduced bias? | Could the patient flow have introduced bias? |
| Concerns about applicability (high, low, or unclear) | Are there concerns that the included patients do not match the review question? | Are there concerns that the imaging methods, its conduct, or its interpretation differ from the review question? | Are there concerns that the target condition as defined by the reference standard does not match the review question? |  |

**Table 4 The extracted raw numbers of the 6 included articles.**

| Author | MRI | | | |  | ^18^FDG PET/CT | | | |
| --- | --- | --- | --- | --- | --- | --- | --- | --- | --- |
|  | TP | FP | FN | TN |  | TP | FP | FN | TN |
| Pengel | 20 | 3 | 23 | 47 |  | 37 | 21 | 6 | 29 |
| Kim | 31 | 5 | 3 | 17 |  | 21 | 4 | 2 | 11 |
| Tateishi | 12 | 10 | 12 | 108 |  | 16 | 5 | 8 | 113 |
| Park, J.S | 5 | 1 | 3 | 23 |  | 8 | 9 | 0 | 15 |
| CHOI | 5 | 1 | 2 | 21 |  | 6 | 21 | 1 | 13 |
| Chen | 1 | 7 | 2 | 6 |  | 9 | 1 | 1 | 5 |
